# Mr. Murray on Small-Pox and Vaccination

**Published:** 1812-09

**Authors:** Charles Murray

**Affiliations:** Bedford-row


*July* 19, 1812.

---

*To the Editors of the Medical and Physical Journal.*

GENTLEMEN,

EVERY philanthropist, and much more every British philanthropist, must deplore the continued ravages of small-pox in this metropolis, where it still sweeps off, in destructive cruelty, an average number of nearly fifty per month; while the disease is almost extinguished in every part of the continent of Europe. This is, however, more a subject of sorrow than surprise, when we see so infectious a malady still disseminated by inoculation and unrestrained intercourse in the very heart of the city; and, unhappily, I fear that, so long as any medical man can reconcile it to himself to promote variolous inoculation by his opinions and practice, there will always be found some prejudiced individuals ready to avail themselves of such a sanction.

Having heard the reiterated allegations respecting the mildness of the inoculated small-pox, and the fact of its average mortality being only one in 300, and from some indeed only one in 500, I must own I was greatly surprised by hearing of several cases of death under inoculation, which occurred within my own knowledge; and this even among those classes where there could be no deficiency in the care of the hapless victims.

This circumstance induced me to extend my inquiries, and, from the best information, I am able to infer that for a considerable time past, the real amount of the mortality by inoculated small-pox, has very greatly exceeded the above computation.

computation. At all events, these instances of fatality are so numerous, as fully to justify this observation; and the records of the practice of those few members of the profession who still take the responsibility of inflicting (if I may so term it) this mortal disease, would doubtless furnish evidence of the fact.

Under such circumstances, surely it is due to justice and humanity, nay I conceive it to be due to their own future tranquillity of mind, that such practitioners should state fairly and openly to every misguided parent, who desires this bane of human nature, the fatal consequences to be apprehended from it; and the suggestion from such authority, where it was least expected, would probably have more influence than the thousand sound arguments with which the zealous medical vaccinist is armed. A *utrum horum* of this kind, fairly put, must certainly have the best effect.

I am aware that these hints will be applicable to a very few of your readers; but, small as the number is, inasmuch as the mischiefs which are occasioned may be boundless, every friend to humanity must wish to see them adopt that forbearance with regard to inoculation, which has for many years past characterized so large a proportion of the profession.

Having said thus much with respect to small-pox, I shall take the liberty to mention one fact in favor of vaccination, which I have not seen hitherto noticed: I mean the impression obviously made by it upon the mortality of small-pox in this metropolis. In 1802, I consider vaccination as fully established in the opinions of nine tenths of the medical profession, and from that period its progress has been steady and continued, though far less than one could wish for the honor of that country which gave birth to the immortal discoverer. Now it appears from the annual bills, that the amount of deaths by small-pox in ten years preceding 1802 was 18,191; and in the following ten years, ending December last, 11,824; making a difference of 6367. This decrease of more than one third in the mortality, must fairly be attributed to the introduction and practice of vaccination alone, and in my humble opinion affords an additional and powerful proof of the incomparable value of that discovery, and of the incalculable benefits to be derived from a zealous and united extension of it. The argument is strengthened also, when it is recollected that the population of the metropolis has greatly increased within the last ten years.

Horrible, therefore, as it is, to reflect on the destruction of 11,824 persons in London alone, by the small-pox, since we possessed a preventive which might ere this have banished the the disease, as it has almost completely done on the immense continent of Europe, it is some consolation to know that the evil has been at least mitigated.

I believe there are many who would be stimulated to increased exertions in promoting vaccination, were they aware of the great amount of our losses by small-pox periodically sustained; and I shall therefore subjoin a table of this year's mortality; and conclude by suggesting a hope that you will devote a few lines to a monthly statement in future.

I am, Gentlemen,
Your most obedient humble Servant,
CHARLES MURRAY.

*Bedford-row,*

*July* 15*th*, 1812.

*Monthly Statement.*

| | | | | |
|---|---|---|---|---|
| 1811. Dec. 17 to Jan. 7, 1812 | 34 | | | 186 |
| Feb. 14 | 52 | April 28 | | 25 |
| Mar. 3 | 50 | May 26 | | 24 |
| —— 31 | 50 | June 2 | | 13 |
| | —— | —— 9 | | 21 |
| | 186 | | | —— |
| Total in half year of twenty-six weeks | | | | 309 |

| | |
|---|---|
| Weekly since June 16 - - | 11 |
| —— 23 - - | 13 |
| —— 30 - - | 26 |
| July 7 - - | 17 |

N. B. The annual Bill of Mortality is brought up to the second Tuesday before Christmas-day in each year.

