# Corrigenda

**Published:** 1812-10

**Authors:** 


### *CORRIGENDA.*

In our last Number, p. 182, in the Monthly Statement, opposite May 26, for 24 read 64.

